# Quantitative Sodium (^23^Na) MRI in Pediatric Gliomas: Initial Experience

**DOI:** 10.3390/diagnostics12051223

**Published:** 2022-05-13

**Authors:** Aashim Bhatia, Vincent Kyu Lee, Yongxian Qian, Michael J. Paldino, Rafael Ceschin, Jasmine Hect, James M. Mountz, Dandan Sun, Gary Kohanbash, Ian F. Pollack, Regina I. Jakacki, Fernando Boada, Ashok Panigrahy

**Affiliations:** 1 Department of Radiology, Children’s Hospital of Philadelphia, 3401 Civic Center Blvd., Philadelphia, PA 19096, USA; 2 Department of Radiology, University of Pittsburgh, Pittsburgh, PA 15260, USA; vkl2@pitt.edu (V.K.L.); paldinomj@upmc.edu (M.J.P.); rcc10@pitt.edu (R.C.); hectjl2@upmc.edu (J.H.); mountzjm@upmc.edu (J.M.M.); ashok.panigrahy@chp.edu (A.P.); 3 Department of Bioengineering, University of Pittsburgh, Pittsburgh, PA 15260, USA; 4 Center for Biomedical Imaging, Department of Radiology, University of Pittsburgh, Pittsburgh, PA 15260, USA; yongxian.qian@nyulangone.org (Y.Q.); reggiejakacki@gmail.com (R.I.J.); 5 Department of Neurology, University of Pittsburgh, Pittsburgh, PA 15260, USA; sund@upmc.edu; 6 Geriatric Research, Educational and Clinical Center, Veterans Affairs Pittsburgh Health Care System, Pittsburgh, PA 15240, USA; 7 Neurological Surgery, University of Pittsburgh, Pittsburgh, PA 15260, USA; gkohanbash@gmail.com (G.K.); pollaci@upmc.edu (I.F.P.); 8 New York University Grossman School of Medicine, New York, NY 10016, USA; fdoboada@stanford.edu; 9 UPMC Hillman Cancer Center, Pittsburgh, PA 15232, USA

**Keywords:** sodium, MRI, brain tumor

## Abstract

Background: ^23^Na MRI correlates with tumor proliferation, and studies in pediatric patients are lacking. The purpose of the study: (1) to compare total sodium concentration (TSC) between pediatric glioma and non-neoplastic brain tissue using ^23^Na MRI; (2) compare tissue conspicuity of bound sodium concentration (BSC) using ^23^Na MRI dual echo relative to TSC imaging. Methods: TSC was measured in: (1) non-neoplastic brain tissues and (2) three types of manually segmented gliomas (diffuse intrinsic brainstem glioma (DIPG), recurrent supratentorial low-grade glioma (LGG), and high-grade glioma (HGG)). In a subset of patients, serial changes in both TSC and BSC (dual echo ^23^Na MRI) were assessed. Results: Twenty-six pediatric patients with gliomas (median age of 12.0 years, range 4.9–23.3 years) were scanned with ^23^Na MRI. DIPG treated with RT demonstrated higher TSC values than the uninvolved infratentorial tissues (*p* < 0.001). Recurrent supratentorial LGG and HGG exhibited higher TSC values than the uninvolved white matter (WM) and gray matter (GM) (*p* < 0.002 for LGG, and *p* < 0.02 for HGG). The dual echo ^23^Na MRI suppressed the sodium signal within both CSF and necrotic foci. Conclusion: Quantitative ^23^Na MRI of pediatric gliomas demonstrates a range of values that are higher than non-neoplastic tissues. Dual echo ^23^Na MRI of BCS improves tissue conspicuity relative to TSC imaging.

## 1. Introduction

Pediatric brain tumors are the most common cause of cancer death in infants and children [1,2]. Conventional (^1^H) MRI is used in the clinical setting and is currently the standard of imaging of pediatric brain tumors and includes multiple sequences, such as T2-weighting, fluid attenuation by inversion recovery (FLAIR), and post gadolinium-based contrast agent (GBCA) T1 sequences, each offering a slightly different tissue sensitivity, but relatively little physiological specificity. Although advanced ^1^H MRI approaches (including diffusion, perfusion and permeability) have been proposed for tumor characterization, precise morphometric measurement can still be inaccurate. There are many tumors that do not demonstrate enhancement after GBCA administration, often negating the benefits of perfusion and permeability MRI, as well as more conventional post-GBCA T1 imaging and even if there is enhancement, it is not necessarily reflective of tumor progression. Just as in true progression, pseudoprogression is characterized by an apparent initial increase in the size of the tumor and contrast-enhancement; however, different from true progression, pseudoprogression is characterized by subsequent tumor shrinkage (regionally or globally). Thus, there are currently no imaging biomarkers that can reliably predict brain tumor progression or response to treatment. More accurate, and physiologically specific, imaging biomarkers are required both for diagnosis and effective monitoring of pediatric gliomas.

More advanced MRI techniques can provide added value in determining tumor response to treatment, but have similar limitations of inconsistency due to complex tumor microenvironments. Sodium concentrations, non-invasively measurable by sodium (^23^Na) MRI, have been shown to be markers of tumor proliferation in animal glioma models and may be useful in monitoring posttreatment responses [3,4]. Increased sodium accumulation has also been seen secondary to other biological processes, including neuroinflammation, compromised mitochondrial metabolism and Na/K ATPase dysfunction [5]. Sodium MRI has been beneficial in evaluation of acute ischemic strokes, multiple sclerosis, amyotrophic lateral sclerosis, migraines, and multiple tumor types, including outside of the central nervous system. Some of these disease processes have demonstrated increased sodium accumulation secondary to neuroinflammation, compromised mitochondrial metabolism and Na/K ATPase dysfunctions. In adult CNS tumors, ^23^Na MRI has provided additional information related to cellular metabolism, complementary to standard proton (^1^H) MRI [6,7].

To our knowledge, ^23^Na MRI has not previously been evaluated in pediatric brain tumors. The primary aim of this study was to evaluate the feasibility of sodium MRI in measuring total sodium concentration (TSC) in both uninvolved brain tissues and three types of pediatric gliomas (DIPG, LGG, HGG). Our secondary aim was to determine the added value of imaging bound sodium concentration (BSC) as measured with the dual echo ^23^Na MRI, with respect to tissue conspicuity. The dual-TE imaging of BSC not only provides improved imaging conspicuity of lesions by suppressing the high CSF signal seen with single TE acquisition, but also is more reflective of intracellular sodium concentration. We quantitated serial changes in TSC and BSC, by utilizing voxel based, parametric mapping techniques.

## 2. Materials and Methods

### 2.1. Participants

Study participants were recruited from patients who were participating in glial-associated antigen peptide vaccine trials through the Pediatric Neuro-Oncology Clinic (RJ) at the Children’s Hospital of Pittsburgh (Supplemental Table S1).

### 2.2. Sodium MR Imaging Acquisition

This study was approved by the institutional ethics committee and informed consent was obtained in all cases.

### 2.3. Sodium MR Imaging Acquisition

^23^Na MRI images were acquired on a 3T Siemens Scanner (TIM Trio, Siemens AG, Erlangen, Germany) with a dual-tuned (^1^H-^23^Na) head volume coil (Advanced Imaging Research, Cleveland, OH, USA). A custom-developed pulse sequence, twisted projection imaging (TPI) [8], was used to acquire total sodium imaging data in all subjects with the following optimized single echo technique: FOV = 220 mm, matrix size = 64 × 64 × 64, voxel size = 3.44 mm (3D isotropic), TE = 0.5 ms, TR = 100 ms, averages = 4, and total acquisition time (TA) = 10 min 38 sec. For a subset of patients, both total sodium and bound sodium imaging was calculated using a two-TE technique which was developed and implemented later in the study [9]: FOV = 220 mm, matrix size = 64 × 64 × 64, voxel size = 3.44 mm (3D isotropic), TE_1_/TE_2_ = 0.5/5 ms, TR = 100 ms, averages = 4, and TA = 10 min 38 sec for each TE.

### 2.4. Post-Processing and Quantitative Sodium MR Imaging

Baseline analysis: intensity of the TE_1_ and short-T_2_ images were linearly calibrated using the CSF region (TSC = 145 mM) and the noise-only background (TSC = 0 mM). Please refer to Appendix A for details about the calculation of sodium signal and additional serial analysis approach. The region of interest (ROI) quantification for normal-appearing grey matter (GM) and white matter (WM) for both TE_1_ and short-T_2_ images was performed. Follow-up sodium images were registered to the anatomical T2 or FLAIR proton images using 6 degrees of freedom rigid body transformation in medical imaging processing, analysis, and visualization (MIPAV). Intensity of all sodium images was normalized relative to that of vitreous fluid. Sodium concentration was then measured in the CSF, vitreous, uninvolved GM, WM, brainstem, and background noise, using uniform ROIs placed in these regions across the participants to ensure sodium measurements were derived from a consistent volume. The primary site of tumors on sodium images were identified and manually segmented with the aid of the regular proton images.

### 2.5. Statistical Analysis

For the primary analysis, analysis of variance (ANOVA) was used to test differences in the total sodium concentration change among the tumor types at the primary lesion ROIs for the three groups. Additional ANOVA was performed among these groups for each of the uninvolved tissue regions (vitreous, CSF, grey matter, and white matter) (Table 1). Linear regression was used to analyze the relationship between total sodium concentration in these regions and the age of the participant. An exploratory univariate regression analysis was performed to correlate serial changes in TSC (mM) with change in tumor volume (mm^3^) as measured on concurrent conventional MRIs. An exploratory agreement analysis between the serial change in TSC (mM) and conventional MRI response was calculated by performing a Cohen’s kappa. Possible sex-based difference in sodium concentration was also examined using a *t*-test on the mean TSC from the aforementioned ROIs. The false discovery rate controlling procedure method was used to correct for family-wise error in multiple comparisons.

## 3. Results

### 3.1. Clinical Characteristics

A total of 26 participants with gliomas were included in the cohort with a median age of 12.0 years; range 4.9–23.3 years; 14 males (a 20+ year old patient was initially diagnosed with a pediatric glioma as a pediatric patient and continued monitoring into adulthood, thus was recruited into this study): DIPG (n = 11, median age of 8.8 years), recurrent supratentorial LGG (n = 6, median age = 16.5 years) and recurrent supratentorial HGG (n = 9, median age = 12.7 years) (Flow chart of participant recruitment displayed in Supplemental Figure S1).

### 3.2. Quantitative Total Sodium Concentration of Normal Brain and Brain Tumors

**Figure 1:**
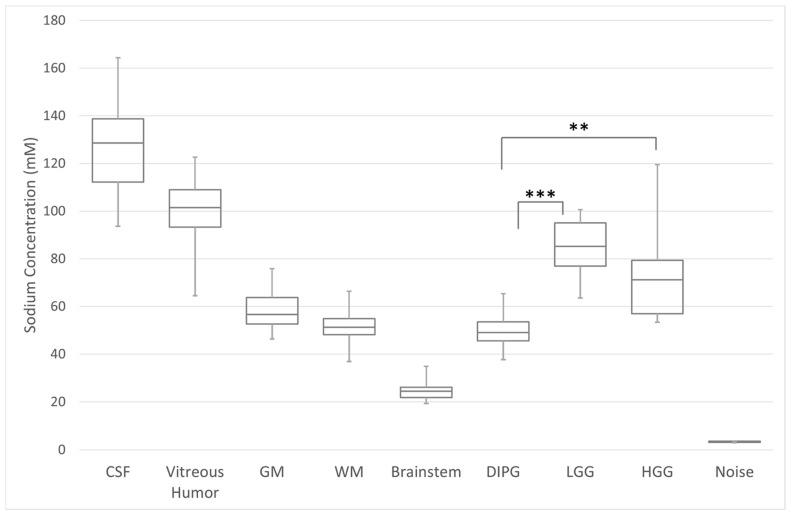
Total sodium concentration (TSC) of the uninvolved cerebral tissue, vitreous humor, and cerebrospinal fluid (CSF), against tumor type. *T*-test comparing tumor grade ** = *p* < 0.05; *** *p* < 0.001. GM = grey matter, WM = white matter, DIPG = diffuse infiltrating pontine glioma, LGG = low-grade glioma, HGG = high-grade glioma.

**Figure 2:**
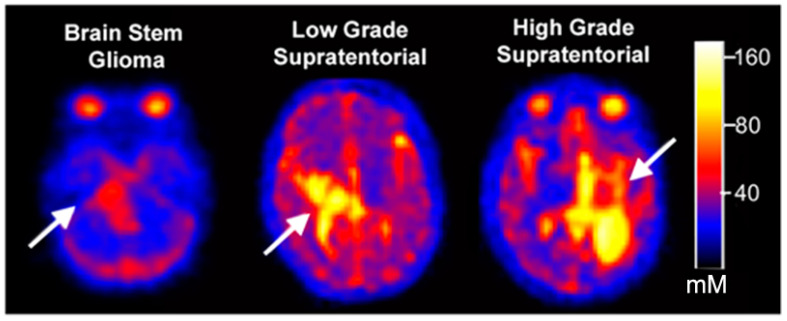
Sodium MRI images demonstrating relative total sodium concentration (TSC) between three different types of pediatric gliomas. There is reduced relative TSC in the pediatric diffuse intrinsic pontine glioma (DIPG) compared to the supratentorial low-grade and high-grade gliomas. There is no difference in relatively high TSC between the low-grade and high-grade gliomas (arrows point to the tumors).

Total sodium concentrations (mean ± SD) were as follows: uninvolved (non-neoplastic) tissue: CSF = 126.6 ± 11.3 mM, vitreous fluid = 99.8 ± 14.9 mM, grey matter = 58.9 ± 5.7 mM, white matter = 52.5 ± 4.7 mM, and normal brainstem = 24.90 ± 4.5 mM (Figure 1 left). An inverse relationship between TSC and age was observed in the GM (R = 0.53207; *p*-value = 0.0035), and WM (R = 0.50744; *p*-value = 0.0061) (Supplemental Figure S2) (Supplemental Table S2). Compared to uninvolved brainstem (measured from portions of the uninvolved pons in participants with supratentorial LGG and HGG), DIPG s/p RT had significantly higher total sodium concentrations (*p* < 0.0001) (Figure 1, right; examples in Figure 2). Among participants with recurrent LGG and HGG, the total sodium concentration in the tumors were compared to their own uninvolved WM and GM measurements. For participants with recurrent supratentorial LGG and HGG, both groups exhibited higher total sodium concentrations compared to their uninvolved WM and GM (*p* = 0.0005 WM and *p* = 0.0011 GM for LGG) and (*p* = 0.0042 for WM and *p* = 0.01781 for GM for those with HGG). (Figure 1, right).

### 3.3. Serial Quantitative Intra-Tumoral Total Sodium Concentration of Pediatric Glioma

**Figure 3:**
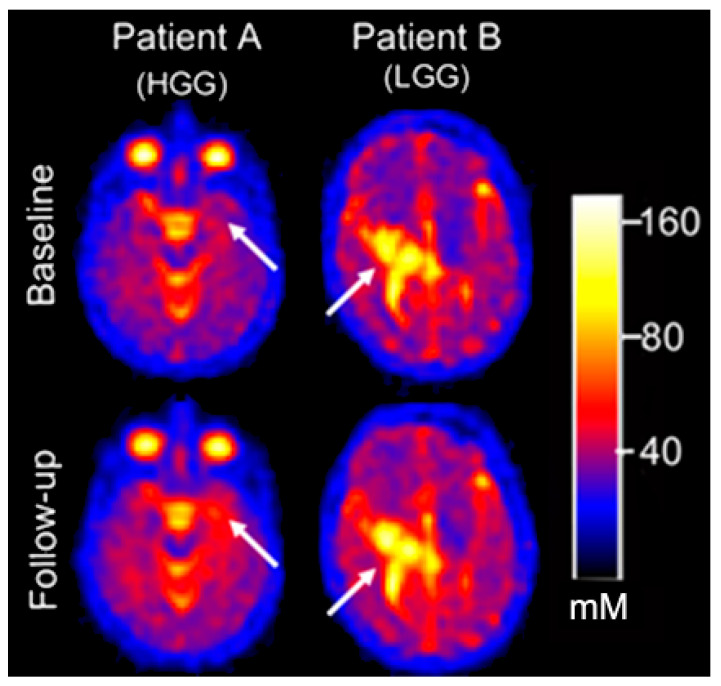
Initial (*top row*) and follow-up (*bottom row*) sodium MRI scans of supratentorial pediatric gliomas with (**A**) increased total sodium concentration (TSC) corresponding to tumor progression in a supratentorial high-grade glioma (arrows) (HGG), and (**B**) no change in TSC corresponding to a stable supratentorial low-grade glioma (arrows) (LGG).

Eight participants underwent serial ^23^Na MRI (total of 20 exams) to measure intratumoral TSC (Figure 3, Supplemental Figures S3 and S4) (Table 2). The eight patients included one with recurrent LGG, four with recurrent supratentorial HGG, and three with DIPG following the completion of RT. Six of these participants underwent only one follow-up sodium scan, whereas two patients with HGG underwent two to four follow-up scans. There was no change in TSC (within ± 1 SD mM) in 7/12 (58.3%) serial exams, increased in 2/12 (16.7%) serial exams, and decreased in 3/12 (25.0%) serial exams. As an exploratory analysis, we observed that although serial % change in TSC did not correlate with % change in concurrent tumor size, serial change in TSC did moderately agree with qualitative multi-modal conventional MRI treatment response assessment in an exploratory analysis (Table 2).

### 3.4. Quantitative Intra-Tumoral Bound Sodium Concentration of Pediatric Gliomas Including Serial Imaging

**Figure 4:**
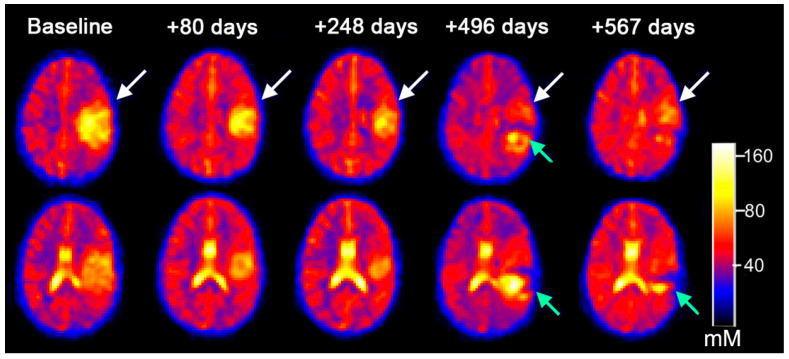
Serial total sodium MRI images at two different axial levels from a participant with supratentorial high-grade glioma treated with immunotherapy. This time series demonstrates a decrease in TSC relative to uninvolved tissue, preceding the eventual lesion size reduction in the tumor (white arrow) as noted by fluid-attenuated inversion recovery (FLAIR) imaging (Supplemental Figure S3). Note, a separate necrotic recurrent lesion (green arrow) also depicted in the sodium vBSC images (Supplemental Figure S5).

**Figure 5:**
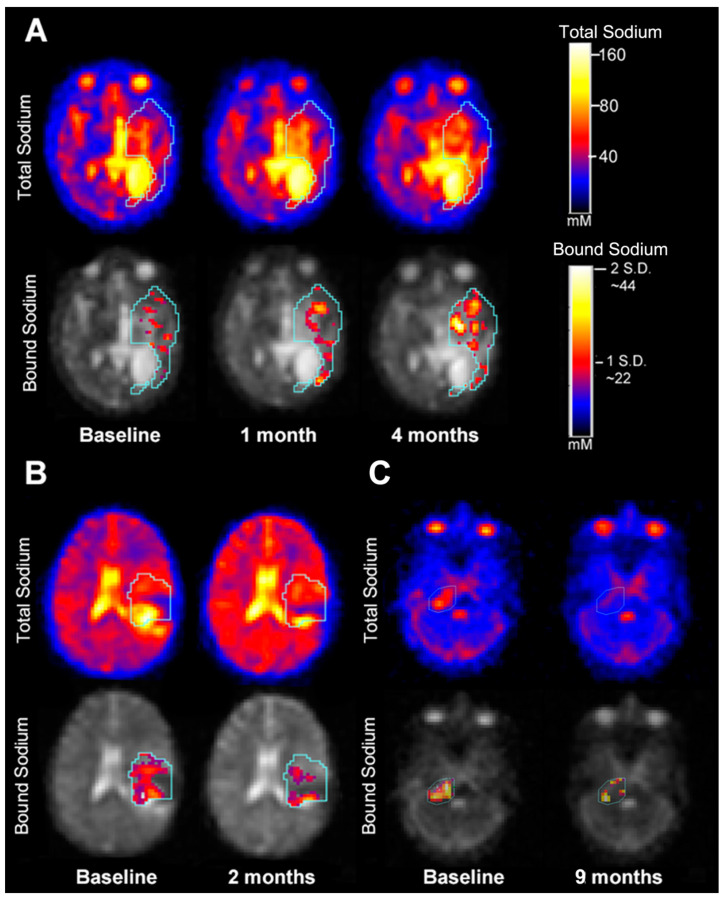
Two-TE sodium MRI scans showing tumor progression in high-grade glioma (Panel (**A**) [pt. ID 4027]), and response to therapy in supratentorial astrocytoma (Panel (**B**) [pt. ID 4010]) and in high-grade glioma (Panel (**C**) [pt. ID 4025). In the tumor regions of the bound sodium images are pixels of vBSC with a value greater than 1 standard deviation (S.D.) from the average vBSC value (~22 mM) over the tumor.

Bound sodium concentration (vBSC) was measured in five patients who underwent the two-TE imaging. We computed vBSC for uninvolved GM (13.8 ± 8.67 mM) and uninvolved WM (14.1 ± 4.49 mM) falls within the accepted range for intracellular sodium in the brain (12–15 mM) (Supplemental Table S3). Particularly for tumors near the ventricular system, the high sodium concentrations within CSF caused significant interference, which was overcome by performing subsequent evaluations using the dual-TE sodium MRI to measure BSC. The dual-TE sodium MRI suppresses the sodium signal within both CSF and necrotic foci, resulting in improved conspicuity of both non-neoplastic and neoplastic tissue located near peripheral cortex and ventricular CSF (Figure 4 and Supplemental Figure S5). Among the group of eight patients that had serial TSC measurements, three patients had serial BSC measurements (Table 2). In one case (Figure 5A), a supratentorial HGG demonstrated no significant change in TSC at one month and four months after baseline sodium imaging, however, BSC did increase and correlated with the conventional imaging of tumor progression. The patient depicted in Figure 5B demonstrated a decrease in TSC and BSC, two months after the baseline sodium MRI, which correlated with a decrease in tumor volume on concurrent conventional MRI. The same patient in Figure 5B demonstrated increased BSC in the region of evolving necrosis (Supplemental Figure S5). Another patient with a HGG (Figure 5C) had a concomitant decrease in TSC and BSC that correlated with a decrease in tumor volume (as measured on conventional MRI) nine months after sodium MRI baseline (Table 2).

## 4. Discussion

This study has demonstrated the feasibility of performing ^23^Na MRI of brain tumors within the pediatric population and was able to distinguish uninvolved brain tissue from neoplastic glial tissue in pediatric patients. The total sodium concentration (TSC) for CSF, vitreous, GM, and WM in our study were similar to what has been reported in the adult literature [7]. We detect high TSC in both recurrent low- and high-grade pediatric gliomas that have distinctive histologies, suggesting that ^23^Na MRI may have less utility in assessing baseline tumor grade in contrast to the serial assessment of therapeutic responses (future studies are warranted). ^23^Na MRI can assess metabolic changes in tissues, e.g., cell integrity and tissue viability with validated repeatability and reproducibility [10,11,12,13,14,15,16,17,18,19]. The sensitivity of sodium imaging stems from the tightly controlled sodium ion homeostasis in healthy tissues which maintains a large concentration gradient between intracellular sodium concentration (ISC) at 10–15 mM and extracellular sodium at 145 mM. Importantly, TSC is elevated in tumors due to increased intracellular sodium (reflecting dysfunction of Na+-K+ pumps on the cell membrane) and/or an increased proportion of extracellular space (changes in cell morphology) [20].

Total sodium concentration in brain tumors can have limitations because of the high sodium signal seen in CSF/necrosis that can mask the intra-tumoral sodium signal related to proliferation. We show the added value of measuring volume-fraction weighted bound sodium concentration (as a proxy of BSC) with dual-TE imaging, which can saturate TSC-high sodium related signal in CSF/necrotic areas, providing better conspicuity of lesions relative to non-neoplastic structures. Dual TE ^23^Na MRI helped mitigate this limitation and potentially enhances its ability to determine heterogeneous treatment responses.

^23^Na MRI is known to be a marker of tumor proliferation in animal glioma models and has shown utility in monitoring posttreatment necrosis and treatment responses in animals [3,4]. As such, we explored the relationship between quantitative serial TSC/BSC measurements and both tumor volume and radiographic response assessment in a small sample of patients. Interestingly, we observed that although serial % change in TSC did not correlate with % change in concurrent tumor size, serial change in TSC did moderately agree with qualitative multi-modal conventional MRI treatment response assessment in an exploratory analysis. In contrast to serial TSC imaging, voxel-based parametric mapping of serial change in BSC did appear to be more reflective of the tumor response assessment within individual patients (in an exploratory analysis). We show that two patients with serial BSC serial changes were concordant with TSC and tumor volume as determined by conventional proton MRI. Studies have investigated the ability to differentiate bound sodium (thought to be reflective of intracellular sodium) and extracellular sodium with various techniques [21,22]. These studies are exploratory and future studies with larger sample sizes are needed to further confirm these results.

The limitations of this study include the small number of heterogeneously treated tumor cases studied, which makes it difficult to compare between the tumor-type groups. For example, the DIPG cohort received radiation therapy 3–4 months prior to obtaining the sodium MRIs, which may cause a treatment related decrease in metabolic activity (lower TSC). There might be a partial volume effect due to the low resolution of the sodium MRI acquisitions, as well as the low SNR of ^23^Na imaging compared to proton imaging. However, with this limitation in mind, the analysis examined intensity of the tumor regions and not the volume measurements. Partial volume effect could potentially affect variation in intensity at the border voxels of the tumor regions, in the context of average quantities, the effect is likely within acceptable limits and will be assessed in future studies.

## 5. Conclusions

In conclusion, we demonstrate the feasibility of quantitatively evaluating ^23^Na by MRI in uninvolved brain tissue of pediatric glioma patients, with similar values to sodium concentrations seen in adults. Diffuse intrinsic brainstem gliomas post-RT and supratentorial gliomas demonstrated total sodium concentrations (TSC) greater than adjacent uninvolved brain tissue. We also show the additional benefit of dual-echo Na MRI (bound sodium concentration) to improve visualization of tumor by distinguishing it from the surrounding tissue and CSF. Future studies are needed to determine the value of ^23^Na MRI in delineating response to treatment in pediatric gliomas.

## Figures and Tables

**Table 1:** ANOVA of ^23^Na of Uninvolved Structures and Pediatric Glioma.

	N-Used	R^2^	Coeff	DoF	F-Value	*p*-Value *
CSF	26	0.0448	14.8171	2/25	0.54	0.5901
Vitreous Humor	26	0.0351	14.2010	2/25	0.42	0.6634
GM	26	0.2611	11.6462	2/25	4.06	0.0308 *
WM	26	0.1803	12.5907	2/25	2.53	0.1016
Tumor	26	0.4926	24.1698	2/25	11.17	0.0004 *

* Corrected for multiple comparison with FDR procedure.

**Table 2:** Serial follow-up sodium (^23^Na) MRI Scans in Evaluation of Pediatric Glioma Immunotherapy.

ID with Scan#	Tumor Total Sodium Concentration TSC, mM			Change in TSC			Conventional Radiographic Response to Treatment at Time of Follow-Up Imaging	Volume (mm^3^)	Volume Change (Absolute in mm^3^/% Change)	Concordant/Discordant (between Changes on Na MRI and Conventional MRI)
	Type	Mean	Std.Dev.	Change	% Change	Direction *				
4002-1	DIPG	54.5052	13.373	6.6723	12	No change	Stable, No tumor progression.	20.8		
4002-2		61.1775	12.1866					25.7	4.9/19%	Concordant
4006-1	DIPG	51.1066	11.9815	2.0928	4	No change	Stable, No tumor progression	41.3		
4006-2		53.1994	12.2965					46.4	5.1/11%	Concordant
4020-1	DIPG	33.7777	5.1005	22.9931	68	Increase	Stable, No tumor progression, increase in necrosis within tumor	24.7		
4020-2		56.7708	14.3001					58.9	34.2/58%	Concordant
4017-1	LGG	101.9751	15.2094	−2.7367	−3	No change	Stable, No tumor progression	85.7		
4017-2		99.2384	15.4893					84.6	−1.1/−1.3%	Concordant
4010-1	HGG	92.5898	12.3779				First serial scan showed decrease in size of the tumor. The second serial scan showed a decrease in size of the tumor The third serial scan showed necrosis with increase in size of the tumor as well as new enhancing lesion The fourth serial scan showed no change in size of the tumor	163.6		
4010-2		73.9051	15.4974	−18.6847	−20	Decrease		120.7	−42.9/−36%	Concordant
4010-3		60.0018	14.2077	−13.9033	−19	Decrease		45.3	−75.4/−166%	Concordant
4010-4		67.2067	20.0919	7.2049	12	No change		43.5	−1.8/−4.1%	Discordant
4010-5		62.0964	13.6286	−5.1103	−8	No change in TSC; decrease in BSC including recurrent lesion between last two dual echo scans		33.8	−9.7/−28.7%	Concordant
4023-1	HGG	49.477	3.7975	7.7313	16	Increase	Interval increase in non-enhancing edema. Infiltrating abnormal signal intensity representing tumor progression	19.2		
4023-2		57.2083	4.3588					39.4	20.2/51.3%	Concordant
4025-1	HGG	47.0821	8.8937	−11.7503	−25	Decrease in TSC and decrease in BSC	Interval increase in size and enhancement of tumor representing recurrence	26.8		
4025-2		35.3318	5.7061					20.4	−6.4/−31.4%	Concordant
4027-1	HGG	77.1108	13.6791			No change in TSC on both serial scans; increase in BSC in recurrent lesion between last two dual echo scans	Progressive increase in size of tumor representing tumor progression	1		
4027-2		81.9984	17.2532	4.8876	6			12.9	11.9/92.2%	Discordant
4027-3		78.9372	18.4989	−3.0612	−4			48.6	35.7/73.5%	Discordant

* determined by a change in +/−1 SD TSC; BSC: volume-fraction weighted bound sodium concentration.

## Data Availability

Not applicable.

## Supplementary Materials

The following supporting information can be downloaded at: https://www.mdpi.com/article/10.3390/diagnostics12051223/s1; Table S1: Participant Characteristics; Table S2: Regression Analysis of *Sodium Intensity* in a given Region of Interest to *Age* of Subject; Table S3: Quantitative Two-TE Sodium Measurements; Figure S1: Flow chart of participant recruitment for sodium MRI studies who were initially recruited as part of a peptide-based vaccine immunotherapy trial; Figure S2: Regression Analysis relating age to average total sodium concentration (TSC) of uninvolved gray matter (GM), uninvolved white matter (WM), and tumor regions. The GM and WM exhibited linear relationship between average TSC and age, while tumor region was not significant; Figure S3: Serial total sodium concentration (TSC) imaging suggesting pseudoprogression. The fluid-attenuated inversion recovery (FLAIR) imaging shows an infiltrative mass in the posterior frontal/parietal region which initially demonstrated increased TSC at baseline. After 4 doses of a vaccine, there is decrease in TSC signal while FLAIR, apparent diffusion coefficient (ADC) and magnetic resonance spectroscopy (white square) are essentially unchanged. At 6 months, there was a clinical concern for progression. However, the sodium continues to show a decreased TSC while the FLAIR signal demonstrates a slight increase with concomitant decreases in ADC and the ratios of Choline/Creatine and Choline/NAA. The sodium MRI and MRS supported pseudoprogression over true progression. Figure S4: Serial TSC in pediatric brainstem glioma [diffuse intrinsic pontine glioma—DIPG]: At baseline, DIPGs have relatively low total sodium concentration (TSC). After therapy, T_2_-weighted image demonstrated an increased hyperintensity within the brainstem. Areas of non-enhancing and enhancing tumor began to evolve in the right inferior brainstem. Sodium MRI revealed a slight increase in TSC within the core of the necrotic region, which was confirmed to be tumor progression. The ADC map demonstrated heterogeneous signal at the cross-sectional timepoint, while a longitudinal analysis of the serial changes (functional diffusion maps) showed spatially and temporally heterogeneous response consisting of areas where there is a clear increase in diffusion (red voxels; co-localizing to the area of increased sodium), decreased diffusion (blue voxels), large areas unchanged (green voxels). The magnetic resonance spectroscopy (MRS) revealed a huge lipid peak, which can be seen both in treatment and tumor related necrosis (red square on bottom posttherapy axial T_2_ is the MRS voxel). Figure S5. Two-TE sodium (^23^Na) MRI of a necrotic recurrent lesion in pediatric supratentorial high-grade glioma. [A] shows a ring enhancing necrotic lesion (red arrowheads); [B] ultra short echo time (TE_1_ = 0.5 ms) and [C] relatively long echo time (TE_2_ = 5 ms) sodium MRI images showing the total and free (extracellular) sodium increasing in the periphery of the lesion (red arrow); [D] relevant region from the bound (intracellular) sodium concentration (vBSC) overlaid on a co-registered structural image, showing the bound sodium increased in the periphery of the necrosis (red and yellow voxels) but decreased in the center (blue voxels), a typical tumor-related cavitation/necrosis. This necrosis was confirmed as a recurrent tumor by the follow up images.

## Abbreviations

ANOVA	analysis of variance
BSC	bound sodium concentration
vBSC	volume-fraction weighted bound sodium concentration
CNS	central nervous system
CSF	cerebrospinal fluid
DIPG	diffuse intrinsic brainstem glioma
FLAIR	fluid-attenuated inversion recovery
GAMs	glioma-associated microglia and monocyte-derived macrophages
GM	gray matter
HGG	high-grade gliomas
IDH	isocitrate dehydrogenase
ISC	intracellular sodium concentration
LGG	low-grade gliomas
MIPAV	medical imaging processing, analysis, and visualization
MRI	magnetic resonance imaging
RAPNO	Response Assessment in Pediatric Neuro-Oncology
ROI	region of interest
RT	radiotherapy
TPI	twisted projection imaging
TSC	total sodium concentration
WM	white matter

## Appendix A. Quantification of Sodium Concentration

An integrated linear calibration on the short-T_2_ image and TE1 image is used to quantify the bound sodium concentration and total sodium concentration, respectively.

(A1) $$C_{total}=C_{fr}\frac{I_{TE1}-{\bar{I}}_{noise,TE1}}{{\bar{I}}_{fr,TE1}-{\bar{I}}_{noise,TE1}}$$

Here *C_total_* is the total sodium concentration, and *C_fr_* is the free sodium concentration known in a voxel of purely free sodium, such as 145 mM in human cerebrospinal fluid. *I_TE1_* is the image intensity at a pixel on TE_1_ image, and *Ī_noise,TE1_* is the mean intensity of noise-only ROI in TE_1_ image. *Ī_fr,TE1_* is T_1_-saturation corrected mean intensity of pixels from a free sodium region of known concentration on the TE_1_ image.

The dual-TE sodium images were used to quantitatively separate between the motion-restricted (or bound) and motion-free (or free) sodium at individual voxels using a two-compartment model, based on their difference in T_2_ relaxation [9]. Sodium nuclei have a spin of 3/2, different from 1/2 for proton (^1^H) nuclei, and thus have bi-exponential decay in T_2_ relaxation: short-T_2_ (~3 ms) decay with 60% of total intensity and long-T_2_ (~15–50 ms) decay with 40% intensity [8]. When sodium ions move fast (relative to Larmor frequency, *f*_0_) and freely (spatially isotropic) in extracellular space in the brain, the sodium nuclear spins only show mono-exponential long T_2_ decay. When moving slowly in intracellular space and being in a gradient electrical field of negatively charged intracellular macromolecules and proteins, the sodium nuclear spins show bi-exponential T_2_ decay. Although accurate quantification of bound sodium concentration (BSC) is challenging due to lack of measurement of volumetric fraction in a voxel, a volume-fraction weighted bound sodium concentration (vBSC) is quantified in this study by following the procedure developed in our previous work [9].

(A2) $$C_{vb}=\frac{C_{fr}}{g\cdot a_{b,S}}\cdot \frac{I_{ST2}-{\bar{I}}_{noise,ST2}}{{\bar{I}}_{fr,TE1}-{\bar{I}}_{noise,ST2}}$$

Here *C_vb_* is vBSC. *I_ST2_* is intensity of a pixel on the short-T_2_ (or bound) sodium image which is the subtraction of TE_2_ image from TE_1_ image, and *Ī_noise,ST2_* is the mean intensity of a noise-only ROI in the short-T_2_ image. g is the gain of short-T_2_ imaging over a range of short T_2_ values of interest, and *a*_b,s_ is 0.6 for the relative intensity of short-T_2_ component of bound sodium.

Serial analysis: Serial sodium images at different time points were compared to the initial Na MRI scan (or to the most recent prior ^23^Na MRI scans for the two cases with multiple follow-up scans) and the difference in total sodium concentration (TSC) or bound sodium concentration (vBSC) for those who underwent dual-TE imaging was calculated. These differences for each participant were then normalized by the standard deviations of their tumor sodium measurements to define individual changes. As an exploratory analysis, TSC changes on serial MRIs were compared to tumor volumes measured on concurrent conventional MRIs. The serial quantitative change in TSC and BSC [i.e., no change, increase, and decrease, based on the unit of one standard deviation (SD)] was correlated with: (1) concurrent tumor volume as measured on T2/FLAIR imaging; and (2) concurrent conventional MRI radiographic response [based on standard clinical interpretation by two board-certified pediatric neuroradiologists which included assessment of tumor size and signal intensity change on T2/FLAIR, post-contrast T1 and diffusion imaging].
