# Supplementary material for: Quantitative Sodium (23Na) MRI in Pediatric Gliomas: Initial Experience
Source: Diagnostics (Basel). 2022 May 13;12(5):1223. doi: 10.3390/diagnostics12051223 (PMC9140048; doi:10.3390/diagnostics12051223)
Supplement: Supplementary file 1 [file diagnostics-12-01223-s001.zip › diagnostics-1663886-supplementary.pdf]

Supplemental Table S1: Participant Characteristics

Tumor Grade	Sex	Age at Diagnosis	Tumor Characteristics	Tumor Location
DIPG	Female	4.9	DIPG	Brainstem
DIPG	Female	5.4	DIPG	Brainstem
DIPG	Female	7.0	DIPG	Brainstem
DIPG	Male	8.8	DIPG	Brainstem
DIPG	Male	8.2	DIPG	Brainstem
DIPG	Male	8.7	DIPG	Brainstem
DIPG	Male	9.8	DIPG	Brainstem
DIPG	Female	10.9	DIPG	Brainstem
DIPG	Female	13.2	DIPG	Brainstem
DIPG	Female	16.7	DIPG	Brainstem
HGG	Male	9.8	Anaplastic astrocytoma	Frontal lobe
HGG	Female	11.5	Glioblastoma	Cerebellopontine angle
HGG	Male	11.8	Glioma	Thalamus
HGG	Female	11.6	Glioma	Occipital lobe
HGG	Male	11.8	Gliomatosis cerebri	Parietal lobe
HGG	Male	12.7	Glioblastoma	Temporal lobe
HGG	Male	14.2	Glioblastoma	Temporal lobe
HGG	Male	17.7	Glioblastoma	Thalamic/intraventricular
HGG	Female	19.8	Oligodendroglioma	Frontal and temporal lobes
HGG	Male	23.3	Glioblastoma	Temporal lobe
LGG	Female	8.3	Glioma (Neurofibromatosis type 1)	Optic pathway
LGG	Female	12.0	Glioma	Optic pathway
LGG	Female	15.8	Glioma	Thalamus
LGG	Male	17.0	Glioma	Temporal lobe and hypothalamus
LGG	Male	17.8	Glioma	Thalamus and basal ganglia
LGG	Male	18.9	Glioma (Neurofibromatosis type 1)	Optic nerve and tectal plate

DIPG = diffuse intrinsic pontine glioma; HGG = high grade glioma; LGG = low grade glioma

Supplemental Table S2. Regression Analysis of *Sodium Intensity* in a given Region of Interest to *Age* of Subject

Tissue ROI	Slope	R^2	R	p-value (FDR $\alpha \leq 0.01$)	Significance ($p \leq \alpha$)	Relation Expected?
CSF	0.11098	0.0008	0.02828	0.8770	No	No
VT (both eyes)	0.37324	0.0165	0.12845	0.5145	No	No
GM	-0.85857	0.2831	0.53207	0.0036*	Yes	Yes
WM	-0.78723	0.2575	0.50744	0.0058*	Yes	Yes
Tumor	1.78362	0.1390	0.37283	0.0507	No	No

* Corrected for multiple comparison with FDR procedure

Supplemental Table S3. Quantitative Two-TE Sodium Measurements

	TSC (mM)	Free Sodium (C_{fr}) (mM)	Bound Sodium (vBSC) (mM)
**GM**	52.4 ± 6.1	38.6 ± 6.17	13.8 ± 8.67 *
**WM**	41.75 ± 3.55	27.65 ± 2.75	14.1 ±4.49
**CSF**	126.64 ±11.32	118.52 ± 12.87	8.12 ± 17.13

*13.80 mM –used as baseline intensity-concentration difference between TSC and free Na; 8.67 mM – used as a relative scaling factor

Supplemental Figures:

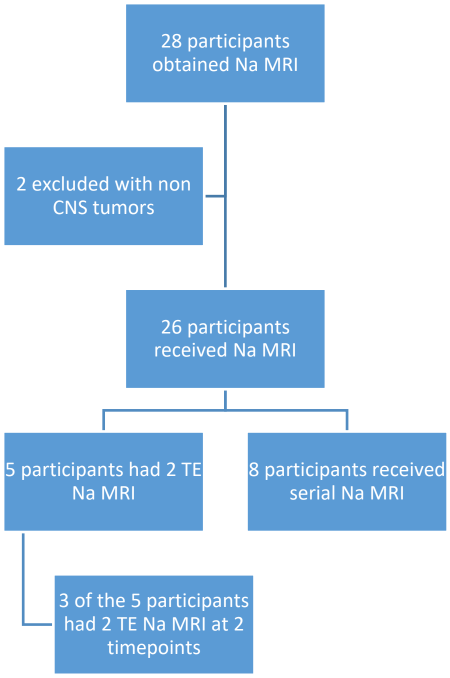

Supplemental Figure S1. Flow chart of participant recruitment for sodium MRI studies who were initially recruited as part of a peptide-based vaccine immunotherapy trial.

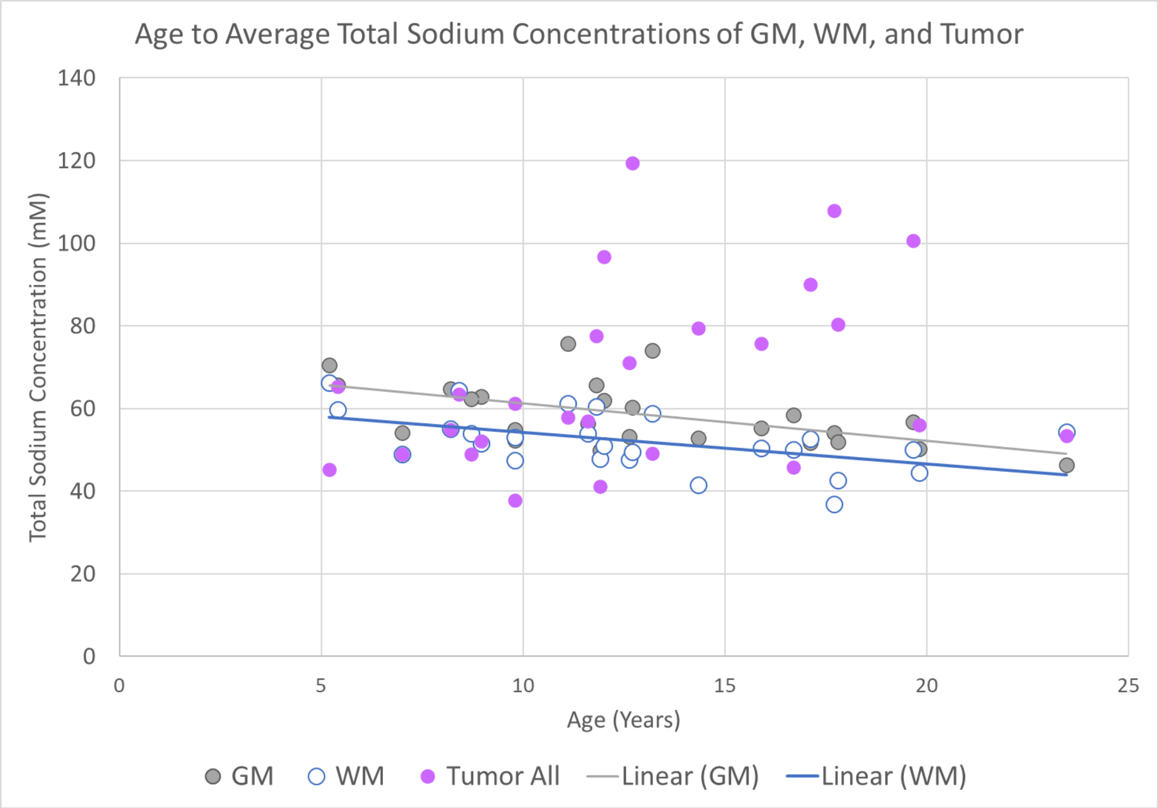

Supplemental Figure S2. Regression Analysis relating age to average total sodium concentration (TSC) of uninvolved gray matter (GM), uninvolved white matter (WM), and tumor regions**.** The GM and WM exhibited linear relationship between average TSC and age, while tumor region was not significant.

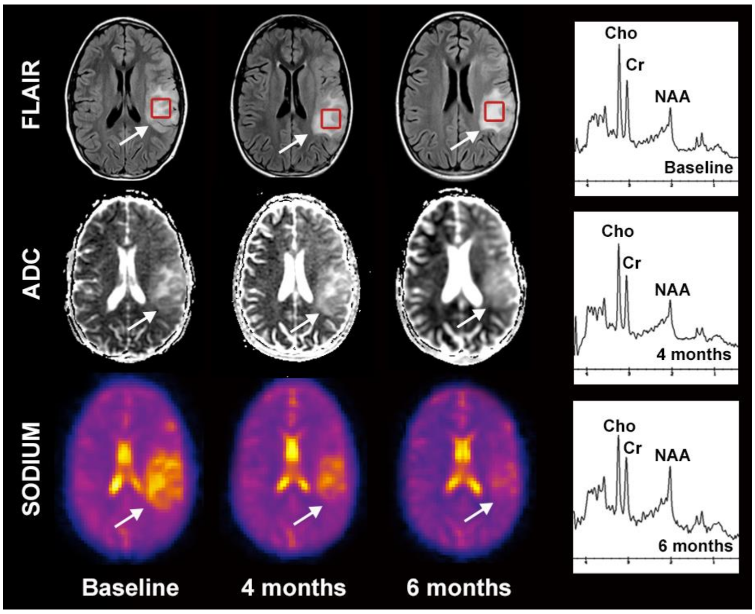

Supplemental Figure S3. Serial total sodium concentration (TSC) imaging suggesting pseudoprogression**.** The fluid-attenuated inversion recovery (FLAIR) imaging shows an infiltrative mass in the posterior frontal/parietal region which initially demonstrated increased TSC at baseline. After 4 doses of a vaccine, there is decrease in TSC signal while FLAIR, apparent diffusion coefficient

(ADC) and magnetic resonance spectroscopy (white square) are essentially unchanged. At 6 months, there was a clinical concern for progression. However, the sodium continues to show a decreased TSC while the FLAIR signal demonstrates a slight increase with concomitant decreases in ADC and the ratios of Choline/Creatine and Choline/NAA. The sodium MRI and MRS supported pseudoprogression over true progression.

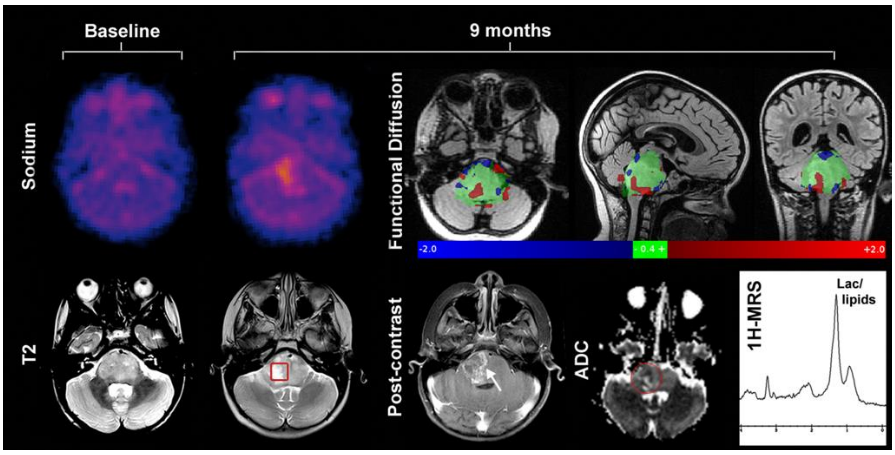

Supplemental Figure S4. Serial TSC in pediatric brainstem glioma [diffuse intrinsic pontine glioma -DIPG]: At baseline, DIPGs have relatively low total sodium concentration (TSC). After therapy, T_2-weighted image demonstrated an increased hyperintensity within the brainstem. Areas of non-enhancing and enhancing tumor began to evolve in the right inferior brainstem. Sodium MRI revealed a slight increase in TSC within the core of the necrotic region, which was confirmed to be tumor progression. The ADC map demonstrated heterogeneous signal at the cross-sectional timepoint, while a longitudinal analysis of the serial changes (functional diffusion maps) showed spatially and temporally heterogeneous response consisting of areas where there is a clear increase in diffusion (red voxels; co-localizing to the area of increased sodium), decreased diffusion (blue voxels), large areas unchanged (green voxels). The magnetic resonance spectroscopy (MRS) revealed a huge lipid peak, which can be seen both in treatment and tumor related necrosis (red square on bottom posttherapy axial T_2 is the MRS voxel).

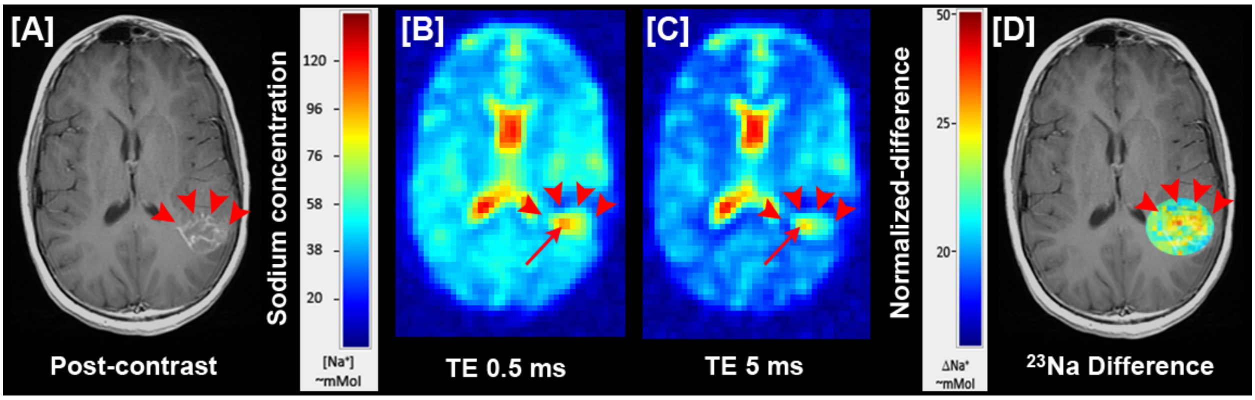

Supplemental Figure S5. Two-TE sodium (^{23}Na) MRI of a necrotic recurrent lesion in pediatric supratentorial high-grade glioma. **[A]** shows a ring enhancing necrotic lesion (red arrowheads); **[B]** ultra short echo time (TE_1 = 0.5 ms) and [**C]** relatively long echo time (TE_2 = 5 ms) sodium MRI images showing the total and free (extracellular) sodium increasing in the periphery of the lesion (red arrow); **[D]** relevant region from the bound (intracellular) sodium concentration (vBSC) overlaid on a co-registered structural image, showing the bound sodium increased in the periphery of the necrosis (red and yellow voxels) but decreased in the center (blue voxels), a typical tumor-related cavitation/necrosis. This necrosis was confirmed as a recurrent tumor by the follow up images.
